# Annotations

**Published:** 1896-11-21

**Authors:** 


### ANNOTATIONS.

### Adulterators and Their Ways.

"How oft the sight of means to do ill deeds make ill deeds done!" A study of some statistics of adulteration, compiled by Dr. Joseph Priestley, the new medical officer of health for Lambeth, and published by him in his first annual report, goes to show that where knavery is exceedingly easy to carry out, and not too certain of detection, about one human being in every five, even among so respectable and mature a class of persons as master tradesmen, is willing to be a knave. We are not going to moralise over this! We simply note it as a fact, for the consideration of moralists, and still more for the instruction of those who are charged with the protection of the health and pockets of the public. By way of illustrating our proposition, that one human being in five is willing to be a knave, if opportunity offers, the following facts are adduced: From the various milk-sellers in Lambeth, 246 samples of milk were procured during the year 1895, and of these 52 were found to be adulterated. From the butter sellers 136 samples were obtained, and 20 of these were adulterated. Of 34 samples of coffee analysed, 9 were impure, and of 12 samples of rum, 3 were adulterated; and so on. All these articles, it will be seen, could be adulterated by any person, however stupid. No skill whatever was required in manipulation; and so the knaves who performed the operation were quite willing to take something surreptitiously out of their neighbours' pockets, so long as the process was easy, and the chance of detection not too great. On the other hand, samples of medicine, of lard, of whiskey, of tomato sauce, and other substances, in which adulteration was difficult or unprofitable, showed that the adulterating process had not been attempted. So much for human nature. Dr. Priestley has not only given his readers food for reflection, but also the means of bringing about practical improvements in the quality of our food stuffs. One other thought may strike the reader, and that is this, that though there are a good many persons who are willing to be guilty of small knaveries, when opportunity offers, there is, after all, a far larger proportion who prefer to respect their own characters and the just laws of their country.

### The Cycle in Spinal Curvature.

An American surgeon, Dr. Kiliani, of New York, has conceived the idea of utilising the bicycle as a means of curing the slighter degrees of lateral curvature of the spine. A considerable time must naturally elapse before it will be possible to express any reliable opinion on the results obtained in a disease the progress of which is notoriously so slow, and whose tendency to recurrence so great, but it is not difficult to see that there is a certain reasonableness in the suggestion. Everyone knows that cases of early scoliosis can usually, by a little care, be put into such a position as, for a time at least, to remove the curvature. The difficulty is that not only do the muscles engaged in maintaining this best position soon get tired, but the rigid maintenance of this position, even for a considerable period, does but little towards preventing the recurrence of the twist so soon as any other posture is taken up. It is not enough to place the patient straight. To do permanent good the muscles which support the spine must be made to go through all their natural movements in this rectified position. Now anyone who watches a cyclist from behind at once becomes aware of the amount of spine movement which is involved in maintaining the balance while the feet are alternately pressing on the pedals, and the suggestion is made that if the patient could but be so placed on the cycle as to bring the spine into its best position, and if then, while in that position, the ordinary exercise of cycling could be indulged in, the result would be far better than can be obtained by any mere postural treatment. The matter is one of very considerable interest. It is quite certain that in a person of normal figure the lowering of one of the cycle handles at once produces a temporary curvature of the spine; and it would seem that in a case of slight scoliosis such a lowering as would produce a curve in the opposite direction must have the effect of rectifying the abnormality. So far we have to do with ordinary rectification by posture. But if when that effect is produced the spine can be made to swing and oscillate, and the muscles to alternately contract and relax in their new position—in fact, if the parts involved can be made to go through all the exercises which so obviously take place in cycling, and to do all that while in their proper, natural position, it is difficult not to believe that the muscles will be thereby strengthened and restored to their normal length, and the spine rendered more capable of retaining the erect position. At the same time, it will always be open to objectors to assert that any benefit which may result is due to improvement in the general health.

### The Dangers of Funerals.

Among the causes of what may be called "minor illnesses," there is hardly one which is so dangerous and so frequent in its incidence in the colder months as attendance at funerals. The wide open doors of the house where the body lies, the generally fireless and draughty rooms, the long slow ride in a hired brougham, which must have its sashes down because of its stuffiness, the sitting uncovered in the damp and chilly church, above all the standing hatless and shivering, after all these preliminaries, in the open air at the graveside whilst the body is being committed to the ground—all these render a chill of a serious kind so common an experience that every man of middle-age protests at every funeral he attends that he will never be present at another until his own turn comes. But an interruption to the continuity of health is really so important to most of us that we may well ask if it is not possible that something should be done to lessen these manifold dangers. It is to be feared that many of these are inseparably connected with all funerals as conducted in this country. But there are at least two things which might be done which would diminish the risk a good deal. The undertaker might be compelled to provide carriages which should be ventilated from the roof, or from some other part which would at least prevent draughts blowing in upon the head and face; and what is much more important, the parish clerk or keeper of the cemetery chapel might and ought to be compelled to have the church or chapel comfortably heated. It is probably here that most of the chills are taken. If some unfortunate sufferer who has been victimised more distressingly than usual would claim damages from one of the rich and prosperous cemetery companies he would confer a great service on the community.

